# Retraction Note: Role of CC-chemokine receptor 5 on myocardial ischemia–reperfusion injury in rats

**DOI:** 10.1007/s11010-023-04897-8

**Published:** 2023-11-25

**Authors:** Bo Shen, Jun Li, Ling Gao, Jieyu Zhang, Bo Yang

**Affiliations:** 1 Department of Cardiology, Renmin Hospital of Wuhan University, Jiefang Road 238, Wuhan, 430060 People’s Republic of China; 2 Cardiovascular Research Institute, Wuhan University, Wuhan, People’s Republic of China; 3 Department of Vascular Surgery, Union Hospital, Tongji Medical College, Huazhong University of Science and Technology, Wuhan, People’s Republic of China

**Retraction Note to: Mol Cell Biochem** 10.1007/s11010-013-1604-z

The Editors-in-Chief have retracted this article. After publication, concerns were raised regarding image irregularities in the figures. Specifically,

- Fig. 4 I/R+Pre and IR+CCR5Ab images appear to overlap.
- Fig. 5 I/R and I/R+Pre images appear to overlap.
- In Fig. 7, the blots in lanes 2 and 5 appear highly similar.

The Editors-in-Chief therefore no longer have confidence in the presented data.

Bo Shen agrees to this retraction. Jun Li, Ling Gao and Bo Yang have not responded to any correspondence from the publisher about this retraction. The publisher has not been able to obtain a current email address for Jieyu Zhang.

